# Distal Renal Tubular Acidosis: A Conundrum of Short Stature, Failure to Thrive, Rickets, and Nephrocalcinosis

**DOI:** 10.7759/cureus.90337

**Published:** 2025-08-17

**Authors:** Pankaj Singhania, Ramamoorthy Ponnusamy, Abhranil Dhar, Ayush Agarwal, Gouranga Santra

**Affiliations:** 1 Endocrinology and Metabolism, Arogyam Health Care, Purulia, IND; 2 General Medicine, Deben Mahata Government Medical College and Hospital, Purulia, IND; 3 Endocrinology and Metabolism, Institute of Post Graduate Medical Education and Research, Kolkata, IND

**Keywords:** growth failure, hypokalemia, renal tubular acidosis, rickets, short stature

## Abstract

Distal renal tubular acidosis (dRTA) is an uncommon disorder marked by defective hydrogen ion excretion in the distal nephron, leading to hyperchloremic metabolic acidosis, persistently alkaline urine, and hypokalemia. Chronic, untreated dRTA in children often presents with growth failure, refractory rickets, and nephrocalcinosis, yet is frequently misdiagnosed. We report a 13-year-old girl with severe stunting, bone pain, proximal muscle weakness, and polyuria for several years. Examination revealed significant short stature, malnutrition, widened wrists, a protuberant abdomen, and genu valgum. X-rays demonstrated classical rachitic changes. Biochemical investigations showed severe hypokalemia (1.3 mEq/L) and metabolic acidosis (pH 7.22, HCO_3_- 7.6 mmol/L) with an inappropriately alkaline urine pH of 6.6. Imaging confirmed medullary nephrocalcinosis. A diagnosis of idiopathic distal renal tubular acidosis (RTA) was made. She was treated with oral alkali and potassium supplements. Over three months, acidosis improved (HCO_3_- 18 mmol/L), potassium normalized (3.2 mEq/L), and X-rays showed healing of rickets. Significant catch-up growth was noted, with a 10 cm height gain and 5 kg weight gain at six months. This case highlights the importance of considering dRTA in children with unexplained growth failure or rickets unresponsive to standard therapy. The classic triad - normal anion gap metabolic acidosis, hypokalemia, and alkaline urine - should prompt evaluation for dRTA. The treatment response with affordable alkali therapy is also highlighted, resulting in miraculous improvement in the patient.

## Introduction

Distal renal tubular acidosis (dRTA), also known as type 1 RTA, represents a clinically significant disorder of renal acid-base homeostasis characterized by the inability of the kidney to excrete hydrogen ions (H+) effectively in the distal nephron. This defect results in hyperchloremic metabolic acidosis with a normal anion gap and persistent alkaline urine (urine pH > 5.5) despite systemic acidosis, associated with electrolyte imbalance, most notably hypokalemia [1,2]. This condition arises from defective H+ secretion or increased H+ back leak in the collecting duct, preventing adequate net acid excretion [1,3]. The clinical consequences include rickets, growth retardation in children, nephrolithiasis, nephrocalcinosis, osteomalacia, polyuria, polydipsia, and chronic hypokalemia [1,3,4].

## Case presentation

A 13-year-old girl born out of a non-consanguineous marriage presented with a history of decreased height and weight gain, proximal muscle weakness, bone pain, and polyuria for the last three to four years. Birth history was insignificant without any history of perinatal complications. There was no history of seizure, tetany, hematuria, delayed dentition, dental abscess, chronic diarrhea, or hearing abnormalities. She had been treated in the past with multiple doses of vitamin D and calcium, which failed to show any improvement. There was no history of other drug intake recently or in the past. 

**Figure 1:**
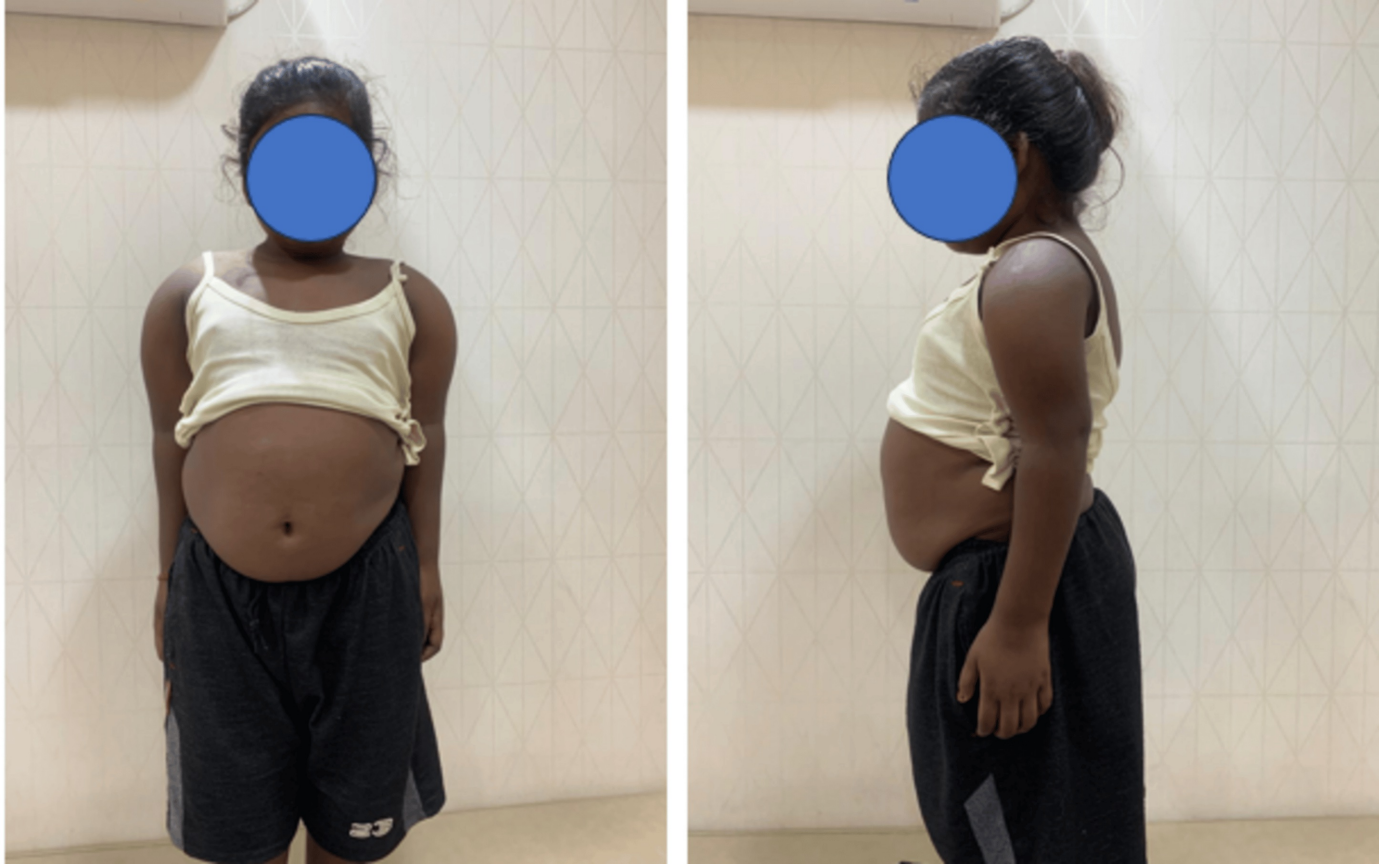
Clinical image of the patient Clinical image of the child showing short stature, protuberant abdomen, and exaggerated lumbar lordosis.

On examination, she had genu valgum, short stature, a protuberant abdomen, and exaggerated lumbar lordosis (Figure 1). She also had widened wrists (Figure 1). She was short and malnourished with a height of 96 cm (-7 standard deviation score (SDS) for her age and sex) and a weight of 18 kg (-3.2 SDS for her age and sex). There were no dental, hearing, or vision-related problems. These were also excluded by clinical examination.

Routine investigations, including examination of peripheral blood smear, were non-remarkable. Urine routine examination was normal, and there was no glucosuria. On biochemical evaluation, serum potassium was 1.3 mEq/L (Table 1). Arterial blood gas (ABG) analysis showed metabolic acidosis (pH-7.228, HCO_3_- 7.6 mEq/L) in the presence of alkaline urine (urine pH 6.6).

**Table 1:** Biochemical investigations at presentation

Parameter	Laboratory value	Reference range
CBC	Hb - 11.2 g/dL	11.5-13
Serum albumin corrected calcium	8.6 mg/dL	8.5-10.5
Serum phosphorous	2.34 mg/dL	4-7
Serum potassium	1.3 mEq/L	3.5-5.5
Serum 25-(OH) vitamin D	27 ng/mL	50-70
Urine calcium: creatinine ratio	0.3	<0.1

**Figure 2:**
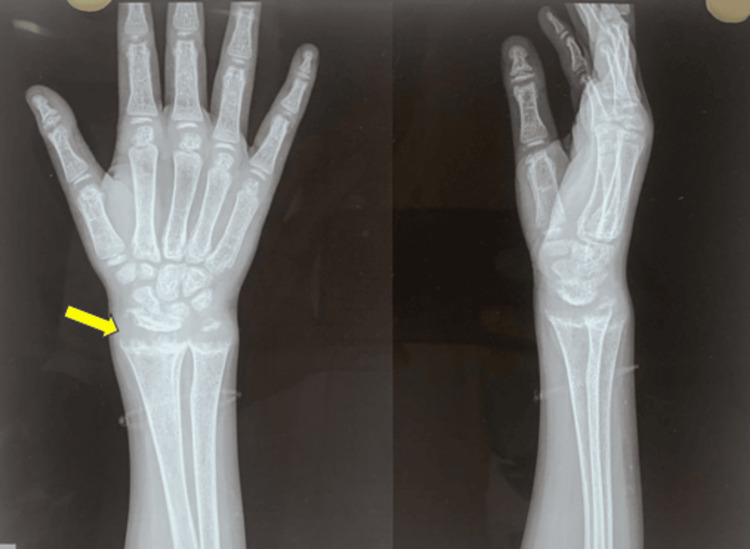
X-ray of right hand X-ray of right hand showing features of rickets in the form of splaying, fraying, and cupping (arrow).

An X-ray of the hand, including wrist joint, showed classical rachitic features in the form of fraying, cupping, and splaying (Figure 2).

**Figure 3:**
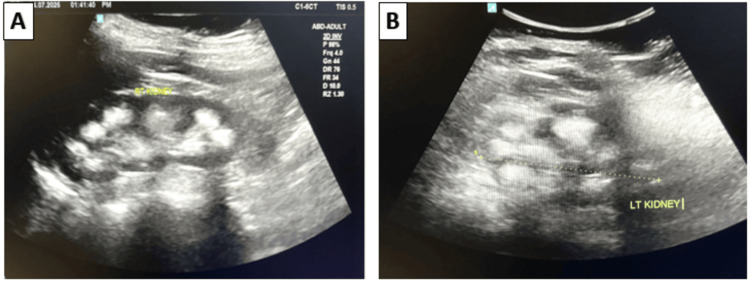
Ultrasonography (USG) of lower abdomen USG of lower abdomen showing multiple echogenic foci in the medullary renal parenchyma of the left (A) and right kidney (B).

On imaging, ultrasonography of the lower abdomen showed multiple echogenic foci noted in the medullary renal parenchyma, suggestive of medullary nephrocalcinosis (Figure 3).

In the presence of metabolic acidosis, alkaline urinary pH (6.6) is suggestive of failure of acidification. The presence of hypokalemia and medullary nephrocalcinosis further gives a clue towards distal RTA. A diagnosis of distal tubular acidosis was made, and attempts were made to arrive at the underlying etiology. However, no secondary autoimmune cause was identified. Therefore, our final diagnosis was rickets due to dRTA (possibly genetic). Genetic cause, though suspected, could not be ruled out due to financial constraints.

Treatment

**Figure 4:**
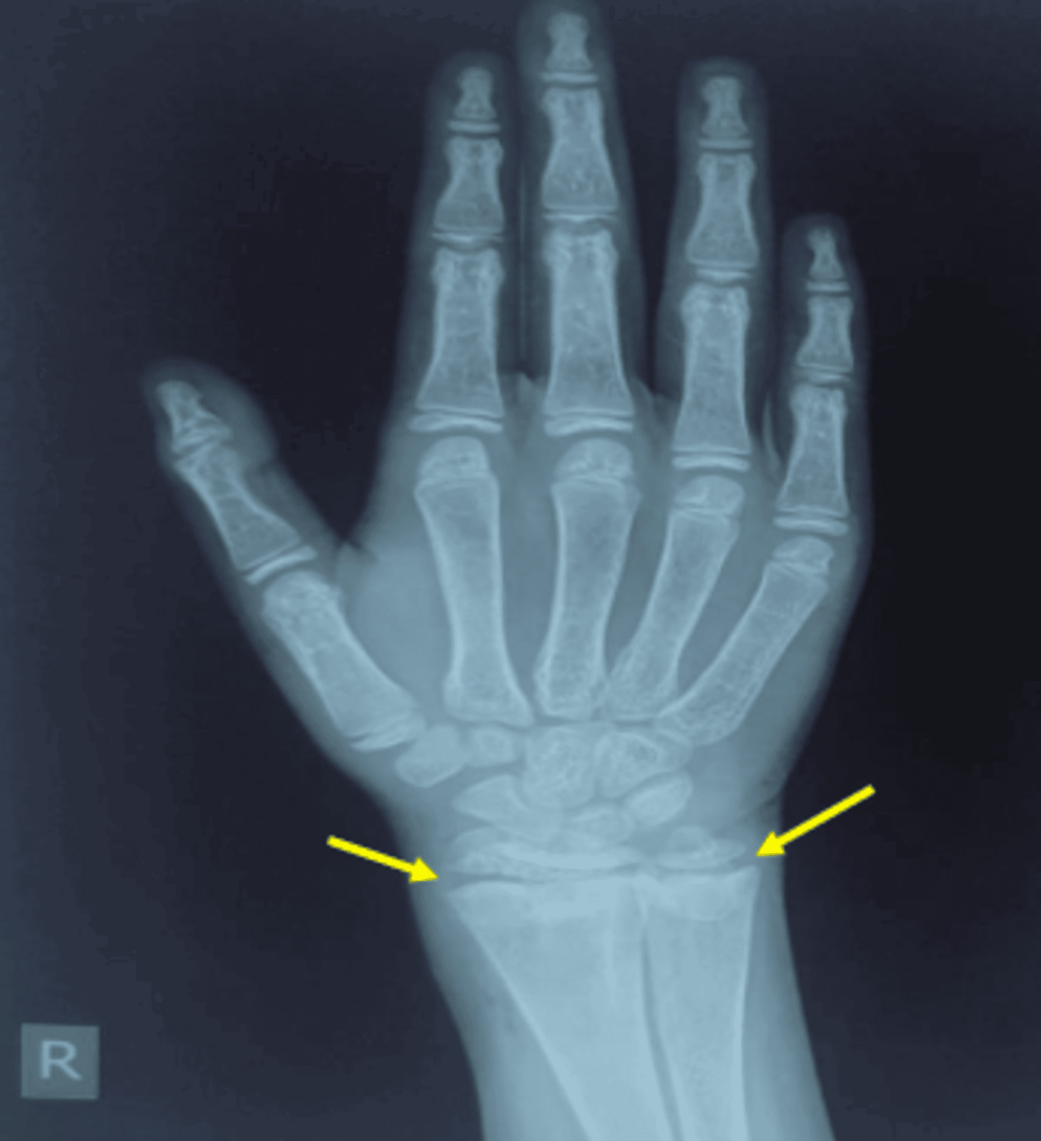
X-ray of left hand X-ray of hand at three-month follow-up showing provisional zone of calcification (yellow arrows).

Patient was initiated on oral alkali therapy at the rate of 2 mEq/kg/day and oral potassium salt at the rate of 2 mEq/kg/day (alkali solution used is composed of potassium citrate 1100 mg and citric acid 334 mg per 5 mL solution, providing 2 mEq of HCO_3_- and 2 mEq of potassium per mL of solution) along with nutritious diet and rich protein and calcium. At follow-up after three months, proximal muscle weakness and bone pain were improved significantly. Repeat ABG showed improvement of acidosis (pH -7.429, HCO_3_- 18). Serum potassium was 3.2 mEq/L (Table 2). An X-ray of the wrist joint showed the appearance of a zone of provisional calcification (Figure 4).

**Table 2:** Biochemical investigations at three-month follow-up

Parameters	Laboratory value	Reference range
Serum potassium	3.2 mEq/L	3.5-5.5
Serum albumin corrected calcium	8.8 mg/dL	8.5-10.7
Serum phosphorous	3.2 mg/dL	4-7
Serum 25-(OH) vitamin D	31 ng/mL	50-70
Urine calcium/creatinine ratio	0.22	<0.16

Height gain of 10 cm and weight gain of 5 kg were observed over six months. She is still following up and improving.

## Discussion

The primary defect in dRTA involves the alpha-intercalated cells of the cortical collecting duct and connecting tubule. Normally, these cells secrete H+ into the tubular lumen via apical H+-ATPase pumps (and to a lesser extent, H+/K+-ATPase exchangers), generating new bicarbonate ions (HCO_3_-) that are returned to the systemic circulation via basolateral anion exchangers (AE1) [2,3,5]. In dRTA, dysfunction occurs due to several potential mechanisms. (1) Defective H+ pump function: Impaired activity or expression of the H+-ATPase pump prevents adequate acidification of the urine. Autoimmune damage (e.g., autoantibodies against H+-ATPase subunits or carbonic anhydrase II) is a key mechanism in acquired forms [5,6]. (2) Increased back-leak of H+: Compromised integrity of the distal tubular epithelium allows secreted H+ to diffuse back into the blood, reducing net acid excretion (e.g., amphotericin B toxicity) [2]. (3) Voltage-dependent defect: Impaired sodium reabsorption in the principal cells (e.g., due to aldosterone deficiency/resistance or ENaC channel blockers like amiloride) reduces the lumen-negative transepithelial electrical potential difference, which is a key driving force for H+ secretion [2,7].

dRTA is frequently linked to autoimmune disorders, with Sjogren syndrome being the most common association. Studies report a prevalence of dRTA (both complete and incomplete forms) ranging from 5% to 25% in Sjogren syndrome patients [4,5]. Systemic lupus erythematosus is another significant, though less common, autoimmune cause [5,8]. The pathogenesis involves immune-mediated tubulointerstitial nephritis, characterized by lymphoplasmacytic infiltrates damaging the tubular cells, particularly the H+-ATPase-rich intercalated cells [3,5]. Rarely, immune checkpoint inhibitors (e.g., PD-1 inhibitors, such as sintilimab and nivolumab) can induce dRTA, likely via similar immune-mediated tubular interstitial nephritis or disruption of adenosine-mediated H+ secretion pathways [9].

Key clinical presentations prompting evaluation include the following. (1) Hypokalemic paralysis: Severe muscle weakness or paralysis due to profound hypokalemia is a frequent initial manifestation, sometimes requiring critical care support [3,4,5]. (2) Nephrolithiasis/nephrocalcinosis: Predominantly calcium phosphate stone formation and medullary calcification occur due to hypercalciuria (from bone buffering of acid), hypocitraturia (citrate reabsorption is enhanced in acidosis), and alkaline urine [2,4,10]. (3) Bone disorders: Chronic acidosis promotes bone demineralization, leading to osteomalacia, rickets in children, osteoporosis, and pathological fractures [4,10,11]. (4) Systemic symptoms: Polyuria, polydipsia, failure to thrive (in children), and symptoms related to underlying autoimmune disease (e.g., xerostomia, xerophthalmia in Sjogren syndrome) [4-6].

In our patient, the presence of chronic systemic acidosis stimulated bone buffering as a compensatory mechanism. Bone carbonate and calcium salts act as a reservoir for buffering excess H+. This leads to dissolution of hydroxyapatite, releasing calcium and phosphate; Inhibition of osteoblast function and stimulation of osteoclast activity.

Shah et al. [12] reported one case having dRTA-associated bilateral renal medullary nephrolithiasis similar to our case. The lithogenic process in distal RTA involves a triad of interrelated urinary abnormalities. (1) Hypercalciuria: Sustained bone resorption releases calcium into the systemic circulation, overwhelming renal resorptive capacity. Additionally, metabolic acidosis directly inhibits calcium reabsorption in the distal tubule and upregulates intestinal calcium absorption through vitamin D-independent mechanisms, resulting in hypercalciuria (typically > 250 mg/24 hours), which provides abundant substrate for crystal nucleation [13,14]. (2) Hypocitraturia: Normally, citrate forms soluble complexes with calcium, inhibiting crystal aggregation. Chronic systemic acidemia enhances the citrate reabsorption in the proximal tubule in dRTA via upregulation of sodium-dicarboxylate cotransporter (NaDC-1). Urinary citrate excretion in dRTA patients is typically <320 mg/day (normal > 450 mg/day), significantly reducing the inhibitory capacity against calcium phosphate precipitation [1]. (3) Persistently alkaline urine: The alkaline environment in urine (typically pH 6.5 to 7.5) dramatically increases the supersaturation of calcium phosphate, particularly when compared to calcium oxalate. [15]

Chronic metabolic acidosis has a profound effect on growth in children. Acidosis directly disrupts the growth hormone (GH), insulin-like growth factor-1 (IGF-1) axis in the growing bone. Research shows that metabolic acidosis induces a state of resistance to GH and IGF-1 in the growth plate cartilage: acidotic conditions reduce GH receptor and IGF-1 receptor expression on chondrocytes and decrease local IGF-1 production [16].

Clinically, growth impairment is a prominent feature of pediatric RTA. One review noted that RTA often presents with growth retardation in childhood and can result in impaired adult height if not treated. Even with treatment, catch-up growth may be incomplete if acidosis was prolonged. For instance, patients with distal RTA diagnosed and treated in infancy tend to achieve near-normal adult height, whereas those diagnosed later often remain shorter than their peers (average final height SDS around -2.0 in one series vs. -1.1 if treated before age 2). This underscores the importance of early intervention [17].

## Conclusions

dRTA in children is a rare but treatable cause of growth failure, rickets, and nephrocalcinosis. Early recognition and initiation of alkali and potassium therapy can significantly reverse metabolic abnormalities and support normal growth. Timely intervention is essential to prevent long-term skeletal and renal complications. Treatment outcomes are extremely rewarding. Catch-up growth and improvement of skeletal deformities start very early in the course of therapy.

## References

[1] Mustaqeem R, Arif A (2023). Renal tubular acidosis. StatPearls.

[2] Palmer BF, Kelepouris E, Clegg DJ (2021). Renal tubular acidosis and management strategies: a narrative review. Adv Ther.

[3] Vasquez-Rios G, Westrich DJ Jr, Philip I, Edwards JC, Shieh S (2019). Distal renal tubular acidosis and severe hypokalemia: a case report and review of the literature. J Med Case Rep.

[4] Queiroz DM, Valenzuela RG, Marinho AW, Santos SS, Silva DO, Dias MD, Cruz LO (2020). Atypical clinical presentation of distal renal tubular acidosis: a case report registered in Amazonas, Brazil. J Bras Nefrol.

[5] Ungureanu O, Ismail G (2022). Distal renal tubular acidosis in patients with autoimmune diseases—an update on pathogenesis, clinical presentation and therapeutic strategies. Biomedicines.

[6] Caretti R, Fiechter C, Babek N, Smith T, Sadiek H (2023). A case report of nearly missed renal tubular acidosis in the setting of Sjögren’s syndrome. Cureus.

[7] Bello CH, Duarte JS, Vasconcelos C (2017). Diabetes mellitus and hyperkalemic renal tubular acidosis: case reports and literature review. J Bras Nefrol.

[8] Malek A, Seyedkaboli S, Batouri A, Khuban AM, Vahedi M (2023). A case report of renal tubular acidosis type 1 without glomerular disease in an adolescent with pediatric-onset systemic lupus erythematosus. Rev Clin Med.

[9] Qiu X, Ren B, Fang L, Dong Z (2023). Anti‑PD1 therapy‑associated distal renal tubular acidosis: a case report. Exp Ther Med.

[10] Bhandarkar A, Varmudy A, Boro H, Bhat S (2025). Renal tubular acidosis: varied aetiologies and clinical presentations: three case reports. World J Nephrol.

[11] Nuransoy Cengi Z A, Yolbaş S, Şahi N İ, Evren B, Özdemi R Z (2022). Type 2 renal tubular acidosis presenting with joint pain: a case report and literature review. Med Int (Lond).

[12] Shah V (2025). Medullary nephrocalcinosis due to type 1 renal tubular acidosis. Radiopaedia.

[13] Fuster DG, Moe OW (2018). Incomplete distal renal tubular acidosis and kidney stones. Adv Chronic Kidney Dis.

[14] Basak RC, Sharkawi KM, Rahman MM, Swar MM (2011). Distal renal tubular acidosis, hypokalemic paralysis, nephrocalcinosis, primary hypothyroidism, growth retardation, osteomalacia and osteoporosis leading to pathological fracture: a case report. Oman Med J.

[15] Awuah Boadi E, Shin S, Yeroushalmi S, Choi BE, Li P, Bandyopadhyay BC (2021). Modulation of tubular pH by acetazolamide in a Ca(2+) transport deficient mice facilitates calcium nephrolithiasis. Int J Mol Sci.

[16] Green J, Maor G (2000). Effect of metabolic acidosis on the growth hormone/IGF-I endocrine axis in skeletal growth centers. Kidney Int.

[17] Liu J, Shen Q, Li G, Zhai Y, Fang X, Xu H (2018). Clinical and genetic analysis of distal renal tubular acidosis in three Chinese children. Ren Fail.

